# The lncRNA ZNF667-AS1 Inhibits Propagation, Invasion, and Angiogenesis of Gastric Cancer by Silencing the Expression of N-Cadherin and VEGFA

**DOI:** 10.1155/2022/3579547

**Published:** 2022-07-01

**Authors:** Chong Yu, Wenqian Chen, Yi Cai, Mingyu Du, Dan Zong, Luxi Qian, Xuesong Jiang, Huanfeng Zhu

**Affiliations:** ^1^Department of Radiation Oncology, Changshu Hospital Affiliated to Soochow University, Changshu No. 1 People's Hospital, Changshu 215500, Jiangsu, China; ^2^Department of Medical Oncology, Changshu Hospital Affiliated to Soochow University, Changshu No. 1 People's Hospital, Changshu 215500, Jiangsu, China; ^3^Department of Radiation Oncology, Jiangsu Cancer Hospital, The Affiliated Cancer Hospital of Nanjing Medical University, Jiangsu Institue of Cancer Research, Nanjing 210000, China

## Abstract

**Purpose:**

Gastric cancer is one of the most common malignancies with high mortality worldwide. It is known that long noncoding RNAs (lncRNAs) play important roles in the pathogenesis of gastric cancer. This study investigates the role of lncRNA ZNF667-AS1 in gastric cancer cells.

**Methods:**

We have applied real-time quantitative PCR (qPCR) to study the levels of ZNF667-AS1 in gastric cancer biopsies and cell lines. The effects of ZNF667-AS1 on the propagation, clonogenicity, metastasis, and angiogenesis of gastric cancer cells were evaluated by calorimetry, colony formation, cell migration, and angiogenesis assays. Western blotting was used to identify the levels of proteins involved in cancer invasion and angiogenesis signaling pathways.

**Result:**

It was found that lncRNA ZNF667-AS1 was downregulated in gastric cancer biopsies. Overexpression of ZNF667-AS1 reduced the propagation, migration, and angiogenesis of gastric cancer cells. Molecular mechanism studies displayed that the high level of lncRNA ZNF667-AS1 promoted the expression of E-cadherin and inhibited the expression of N-cadherin and VEGFA, leading to the inhibition of the proliferation, migration, and angiogenesis of gastric cancer cells.

**Conclusion:**

As a tumor suppressor gene, lncRNA ZNF667-AS1 significantly hinders the propagation, metastasis, and angiogenesis of gastric cancer cells by promoting the expression of E-cadherin and inhibiting the expression of N-cadherin and VEGFA. Therefore, lncRNA ZNF667-AS1 could play a synergistic therapeutic role by targeting tumor cells and vascular endothelial cells, which represents a new therapeutic scheme for novel therapeutics of gastric cancer.

## 1. Introduction

Gastric cancer is one of the most common malignancies worldwide, with high mortality [1, 2]. Because of the lack of typical clinical symptoms in the early stage, gastric cancer is often diagnosed at a late stage with abdominal or distant metastasis, resulting in the loss of the best time of intervention. In recent years, with the improvement of basic examination and related treatment technologies, the early detection and treatment of gastric cancer have improved significantly [3]. However, the effectiveness of the current chemotherapeutic drugs is not ideal. Drugs such as ramucirumab [4, 5] are currently used as antiangiogenesis agents that inhibit tumor angiogenesis. Because of certain adverse reactions and resistance caused by targeted drugs, there is no obvious improvement in the overall survival rate. Therefore, it is of great significance to seek new targeted therapy targets for the clinical treatment of gastric cancer.

Long noncoding RNAs (lncRNAs) are RNAs with more than 200 nucleotides with no protein-coded function. The lncRNAs play crucial regulatory roles in numerous diseases through multiple pathways, especially in malignant tumors like esophageal, prostate, and liver cancers [6–9]. Accumulating data have shown that gastric cancer is usually accompanied by a dysregulated expression of various lncRNAs (such as lncRNA CRNDE, lncRNA AK023391, and lncRNA CASC2), and these lncRNAs regulate tumor metastasis, angiogenesis, and drug resistance through various mechanisms [10–12]. Therefore, lncRNAs can be used as biomarkers for monitoring tumor metastasis and as therapeutic targets for inhibiting tumor progression. Furthermore, the abnormal expression of lncRNAs can serve as a molecular indicator for the identification, treatment, and forecast of gastric cancer. For example, lncRNA PTCSC3 is downregulated in plasma of patients with gastric cancer and is considerably different in samples from healthy controls. During the follow-up period, the general endurance proportion of patients with low amounts of blood plasma lncRNA PTCSC3 was significantly reduced. Thus, this gene can serve as a molecular indicator for the treatment and prognosis of these types of cancers [13].

The lncRNA ZNF667-AS1 is an intracellular zinc finger protein 667-antisense RNA 1, which controls the incidence and progression of various maladies, including cancer. For example, in spinal cord injury, the expression of lncRNA ZNF667-AS is downregulated, and by inhibiting the JAK/STAT signaling pathway, it inhibits the occurrence of inflammation and promotes recovery [14]. The lncRNA ZNF667-AS1 can also alleviate rheumatoid arthritis by regulating miR-523-3p through inhibiting the JAK/STAT signaling pathway [15]. In addition, lncRNA ZNF667-AS1 inhibited the EMT (epithelial-mesenchymal transition) process of oral squamous cell carcinoma by changing the expression of TGF-*β*1 [16]. The lncRNA ZNF667-AS1 serves as a possible diagnostic and molecular predictor in glioma [17]. The above results indicate that lncRNA ZNF667-AS1, as an important intracellular lncRNA, regulates the occurrence, expansion, and early diagnosis of cancer cells. However, the effect and mechanism of lncRNA ZNF667-AS1 on the progression of gastric cancer, its metastasis, and angiogenesis remain unclear. Here, we investigate the expression and function of lncRNA ZNF667-AS1 in patients with gastric cancer and its role in diagnosis and targeted therapy.

## 2. Materials and Methods

### 2.1. Tissue Samples and Sources

The research was approved by the ethics committee of our research institution. Twenty patients who were diagnosed with gastric cancer and underwent surgery in the hospital from May 2019 to January 2021 were included in the study. The patients did not receive radiotherapy or chemotherapy before surgery. The gastric cancer and its paracancerous tissues (more than 5 cm away from the tumor) were collected. Inclusion criteria were as follows: gastric cancer confirmed by pathological or cytological examination and TNM stages IIb–IV and Karnofsky function score ≥70 points, expected to survive more than 3 months. Exclusion criteria were as follows: patients with autoimmune system syndromes, patients with malicious cancers in other parts, and individuals with severe abnormalities of important organs. All participants submitted their informed consent.

### 2.2. Cell Culture and Transfection

The SGC-7901, HGC-27 (gastric cancer cell lines), and gastric epithelial cells GES-1 were cultivated in RPMI 1640, and AGS cells were cultured in F12 K medium (supplemented with 10% FBS and 100 U/ml of penicillin and streptomycin). Cells were cultured at 37°C with 5% CO_2_. After the cells reached confluence, they were digested and passaged. Cells in the logarithmic growth phase were used for experiments.

### 2.3. Real-Time Fluorescent Quantitative PCR

Clinical tissue samples and cells in the logarithmic growth phase were collected. The TRIzol reagent was used to isolate whole RNA from tissues or cells following the manufacturer's protocol. A NanoDrop was applied to detect the concentration of extracted RNA. cDNA was prepared by an RT-PCR kit (Merck, America). 2 *μ*L of reverse transcription product was taken as a template. *β*-Actin was used as an internal reference, and the SYBR Green PCR Master Mix kit was used for qPCR detection. The reaction program was 5 min at 95°C, followed by three-step reactions at 95°C/30 s, 60°C/30 s, and 72°C/10 s, for 40 cycles. After the reaction, the relative quantitative 2-^ΔΔ^Ct method was used for analysis.

### 2.4. MTT Assay for Cell Viability

The transfected gastric cancer cells were collected and seeded into 96-well plates. After culturing at different time intervals, 20 *μ*L of MTT mix was added to each well, and the cells were hatched at 37°C for 4 hours. The 96-well plates were taken out, and the supernatant was removed. Then, 150 *μ*L of DMSO was added, mixed thoroughly, and reacted for 10 min. The OD value (optical density) of each well was assessed at 450 nm by a microplate reader.

### 2.5. Colony Formation Assay

The transfected cultured gastric cancer cells were processed with trypsin, gathered at 940 × *g* for 3 min, and the supernatant was discarded. 5 mL of sterile PBS solution was inserted. The precipitate was washed and centrifuged again. The cell concentration was adjusted to 500 cells/mL, and the single-cell suspension was added into a 6-well plate for colony formation experiments. The cells were divided into control, pcDNA3.1, and ZNF667-AS1 groups, and the culture medium was replaced at 3 days. After 14 days, the colonies were stained with crystal violet and the number was calculated.

### 2.6. Assessment of In Vitro Cell Migration

The superior cavity of each transwell was covered with a layer of Matrigel to simulate the in vivo environment. The cell suspension in the exponential cell growth was gathered and washed away three times with ice-cold PBS buffer. The cell concentration was set to 2 × 10^5^/mL. 20 *μ*L of these cells was put into the superior cavity of the transwell, and then, 500 *μ*L of DMEM complete medium was added to the lower chamber of the transwell. The chamber was hatched at 37°C overnight, fixed with formaldehyde, dyed with crystal violet, and visualized under a light microscope. The cells were counted under the x200 field of view for 5 randomly chosen fields. The study was done in triplicates and the average score was calculated.

### 2.7. Endothelial Cell Tube Formation Assay

The Matrigel was placed with the medium without fetal bovine serum in the refrigerator for 12 hours until it solidified. The medium and Matrigel were mixed in a ratio of 1 : 2, and 250 *μ* of diluted Matrigel was added. Matrigel was put into the 24-well plate and then hatched at 37°C for 1 h. The Matrigel was observed to coagulate into blocks. The density of the HUVEC suspension was adjusted to 3 × 10^6^/mL; 0.1 mL of cells was added dropwise to a 24-well cell culture plate prelaid with Matrigel and finally placed in a 37°C cell for 1 h. The formation of tubules was observed and photographed under a microscope.

### 2.8. Western Blot

Western blot was performed as previously described [18]. After collecting the cells of each group, they were lysed with RIPA buffer to extract the total protein, and the cytoplasmic nuclear protein extraction kit was applied to isolate the cellular protein. The BCA method allowed the determination of protein concentration. 50 *μ*g of the histone samples was taken for SDS-PAGE electrophoresis, transferred to a PVDF membrane, diluted with TBST antibody diluent, and incubated overnight. HRP-labeled secondary Abs were used for incubation with the samples at room temperature for 2 hours. After rinsing with PBS buffer, the membrane was developed using ECL luminescence solution, exposed with a BioRad gel imaging system, and pictures were analyzed.

### 2.9. Statistical Analysis

Data are presented as mean ± standard deviation (S.D). The SPSS 19.0 (SPSS) software was used to conduct all statistical data. Student's *t*-test allowed evaluation of the differences among variables. *P* values ˂0.05 were considered statistically significant.

## 3. Result

The lncRNA ZNF667-AS1 is expressed at a low level in gastric cancer samples and cell lines.

**Figure 1:**
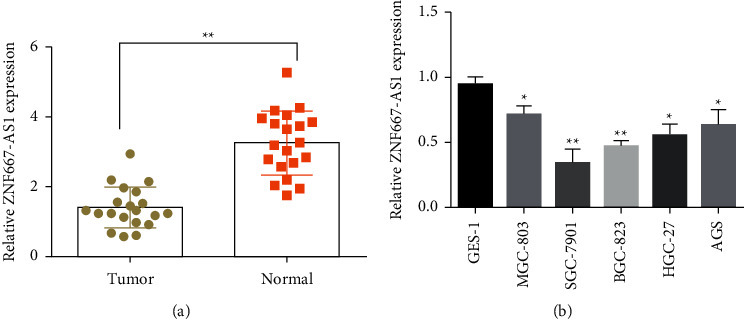
Low expression of ZNF667-AS1 in gastric cancer biopsies (a) and cell lines (b), ^*∗∗*^*p* < 0.01.

To examine the expression of lncRNA ZNF667-AS1 in gastric cancer, we used real-time fluorescence quantitative PCR to examine lncRNA ZNF667-AS1 transcription. Our findings confirmed that lncRNA ZNF667-AS1 levels were lower in gastric cancer biopsies than in paracancours tissue (Figure 1(a), *p* < 0.05). The lncRNA ZNF667-AS1 levels were not associated with gender, age, and body mass index (BMI) (*p* > 0.05), whereas they were linked with TNM stage (*p*=0.0410) and metastasis in the lymph nodes (*p*=0.0383) (Table 1).

The expression levels in MGC-803, SGC-7901, BGC-823, HGC-27, and AGS, as well as in GES-1, were measured. The results displayed that compared with normal epithelial cells from the stomach, lncRNA ZNF667-AS1 was poorly expressed in gastric cancer, and its transcription in the SGC-7901 cell line was the lowest (Figure 1(b), *p* < 0.05). Therefore, we selected SGC-7901 cells for research in the follow-up study.

### 3.1. Overexpression of lncRNA ZNF667-AS1 Inhibits the Propagation of Gastric Cancer Cells

**Figure 2:**
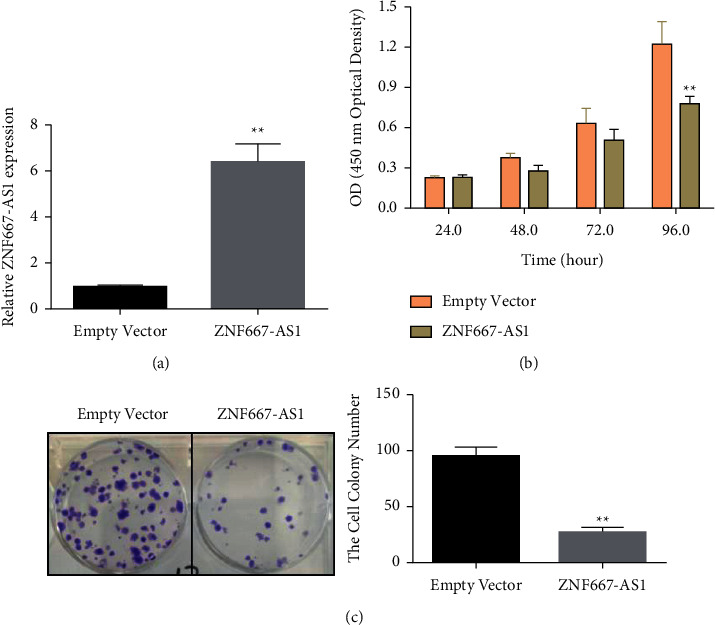
Overexpression of lncRNA ZNF667-AS1 constrains the propagation of gastric cancer cells. (a) Real-time PCR detection of lncRNA ZNF667-AS1 expression levels after transfection. (b) MTT assay detecting the overexpressed ZNF667-AS1 on the propagation ability of gastric cancer cells. (c) Colony formation assay identifying the consequence of lncRNA ZNF667-AS1 overexpression on the colony formation ability of gastric cancer cells, ^*∗∗*^*p* < 0.01.

We next explored the biological function of lncRNA ZNF667-AS1 in these tumor cells and found that it was poorly expressed in gastric cancer SGC-7901 cells. The overexpression of lncRNA ZNF667-AS1 by lentivirus considerably augmented the expression of intracellular lncRNA ZNF667-AS1 (Figure 2(a)). The results of the MTT assay indicated that compared with the blank vector control group (empty vector), upregulation of lncRNA ZNF667-AS1 significantly inhibited the propagation of gastric cancer cells (Figure 2(b)). The clonogenicity experiment showed that upregulation of lncRNA ZNF667-AS1 considerably hindered the colony formation of gastric cancer cells compared with the blank vector group (empty vector) (Figure 2(c)). These data demonstrated that ZNF667-AS1 significantly affected the propagation ability of gastric cancer cells.

### 3.2. Upregulation of lncRNA ZNF667-AS1 Constrains Metastasis and Vascular Survival of Gastric Cancer Cells

**Figure 3:**
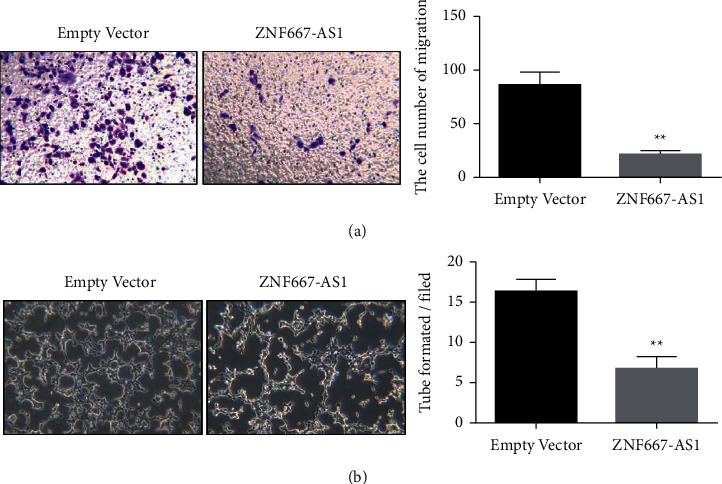
Overexpression of lncRNA ZNF667-AS1 impedes the metastasis (a) and angiogenesis (b) of gastric cancer cells, ^*∗∗*^*p* < 0.01.

The result of ZNF667-AS1 overexpression on inhibiting the migration and angiogenesis of gastric cancer cells was further examined. The results of the transwell assay proved that compared with the blank vector cohort (empty vector), overexpression of lncRNA ZNF667-AS1 considerably constrained the migration ability of gastric cancer cells (Figure 3(a)). Angiogenesis experiments showed that lncRNA ZNF667-AS1 upregulation expressively repressed the ability of angiogenesis compared with the blank vector group (empty vector) (Figure 3(b)). These data show that lncRNA ZNF667-AS1 upregulation significantly affects the metastasis and angiogenesis of gastric cancer cells.

The molecular mechanism of inhibition of passage and angiogenesis of gastric cancer cells by overexpression of lncRNA ZNF667-AS1 was further detected by Western blot. Data showed that lncRNA ZNF667-AS1 upregulation significantly promoted the expression of E-cadherin in gastric cancer cells and inhibited the expression of N-cadherin and VEGFA.

## 4. Discussion

Current data demonstrate that angiogenesis is required for the development and metastasis of gastric cancer, and angiogenesis is regulated by a series of angiogenetic factors and inhibitory factors, which affect the prognosis of patients. Therefore, inhibiting angiogenesis can hinder malignant tumor development and improve the prognosis of patients [19, 20]. The lncRNAs are noncoding RNAs that are longer than 200 nucleotides without open reading frames and cannot encode proteins [21]. Earlier data show that lncRNAs are oddly conveyed in a diversity of malignant cancers and participate in the biological behaviors of tumor cells like propagation, invasion, migration, and angiogenesis [22, 23]. The lncRNA ZNF667-AS1 is a recently recognized noncoding RNA with significant controlling functions, and it is expressed at a low level in a variety of tumors, such as colorectal cancer, melanoma, and leukemia [24–26]. There are few studies on the role of ZNF667-AS1 on the tumorigenic characteristics of gastric cancer. This study revealed that ZNF667-AS1 is expressed at a low level in gastric cancer biopsies and cell lines, and its overexpression considerably constrains the propagation, migration, and angiogenesis of gastric cancer cells. Molecular mechanism studies displayed that overexpression of lncRNA ZNF667-AS1 promotes the expression of E-cadherin and inhibits the expression of N-cadherin and VEGFA.

Accumulating evidence suggests that lncRNAs control tumorigenesis and cancer development. They act as tumor suppressors or oncogenes to influence cancer development through numerous pathways like the regulation of transcription at the epigenetic and posttranslational levels [6]. For example, lncRNA SNHG15 is upregulated in gastric cancer, and silencing SNHG15 constrains the propagation, migration, and invasion and promotes apoptosis of gastric malignant cells [27]. The lncRNA DLEU2 is upregulated in gastric cancer cells. Its level is significantly correlated with the pathological grade and TNM stage of patients. Cell function experiments show that downregulation of DLEU2 constrains cell propagation and metastasis [28]. Our results showed that ZNF667-AS1 was downregulated in gastric cancer tissues and cell lines. Its overexpression considerably constrained the propagation and migration of gastric cancer cells via silencing N-cadherin and upregulating E-cadherin. Our findings are in correlation with previous reports for cervical cancer where lncRNA ZNF667-AS1 hindered tumor cell propagation and metastasis [29]. Overexpression of lncRNA ZNF667-AS1 in colorectal cancer inhibited cell propagation, migration, and metastasis by controlling the ANK2/JAK2 signaling pathway [24].

VEGF is one of the most important regulators of angiogenesis, which regulates tumor angiogenesis by binding to VEGFR2. Tumor angiogenesis, growth, and metastasis could be shut down once the VEGF/VEGFR2 signaling pathway is blocked [30]. For example, PVT1 stimulated angiogenesis by triggering the STAT3/VEGFA signaling pathways in gastric cancer [31]. The lncRNA TUG1 enhances the malignant behavior of gastric adenocarcinoma by regulating miR-29c-3p to upregulate VEGFA [32]. This study shows that overexpression of lncRNA ZNF667-AS1 can significantly downregulate VEGFA in gastric cancer cells and constrain the angiogenesis ability of vascular endothelial cells. However, the underlying mechanisms of ZNF667-AS1 in tumor suppression are yet unknown, which is determined by the intracellular role of lncRNA ZNF667-AS1. Therefore, the role of lncRNA ZNF667-AS1 in gastric cancer cells warrants further study to predict its possible regulatory molecules.

In conclusion, this study revealed that ZNF667-AS1 is expressed at a low level in gastric cancer cells, and overexpression of ZNF667-AS1 constrains the propagation, migration, and angiogenesis of gastric cancer cells. Molecular mechanism studies indicated that overexpression of lncRNA ZNF667-AS1 promotes E-cadherin and inhibits N-cadherin and VEGFA transcription. Therefore, lncRNA ZNF667-AS1 could play a synergistic therapeutic role by targeting tumor cells and vascular endothelial cells and represent a new therapeutic scheme for the clinical management of gastric cancer.

## Figures and Tables

**Figure 4:**
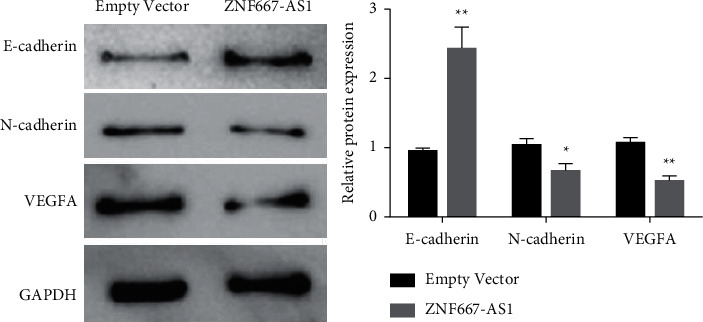
Western blot detection of lncRNA ZNF667-AS1 upregulation hinders the propagation and angiogenesis of gastric cancer cells, ^*∗*^*p* < 0.05, ^*∗∗*^*p* < 0.01.

**Table 1:** Correlation analysis of lncRNA ZNF667-AS1 levels and clinical features of patients with gastric cancer.

Clinical characteristics	*n*	lncRNA ZNF667-AS1 expression	*t*/*F*	*P*
Gender				
Male	12	1.83 ± 0.94	0.4078	0.6883
Female	8	1.66 ± 0.87		

Age				
>60	15	1.72 ± 1.05	0.7054	0.4896
≤60	5	2.11 ± 1.14		

BMI				
>24 kg/m^2^	9	1.56 ± 0.76	0.8827	0.3891
≤24 kg/m^2^	11	1.92 ± 1.01		

TNM stage				
IIb	10	2.21 ± 0.89	3.878	0.0410
III	5	1.46 ± 0.83		
IV	5	0.98 ± 0.69		

Lymph node metastasis				
Yes	13	1.29 ± 0.61	2.235	0.0383
No	7	2.15 ± 1.13		

## Data Availability

All data used to support the findings of this study are available from the corresponding author upon request.
